# Impact of Strategically Located White Matter Hyperintensities on Cognition in Memory Clinic Patients with Small Vessel Disease

**DOI:** 10.1371/journal.pone.0166261

**Published:** 2016-11-08

**Authors:** J. Matthijs Biesbroek, Nick A. Weaver, Saima Hilal, Hugo J. Kuijf, Mohammad Kamran Ikram, Xin Xu, Boon Yeow Tan, Narayanaswamy Venketasubramanian, Albert Postma, Geert Jan Biessels, Christopher P. L. H. Chen

**Affiliations:** 1 Department of Neurology, Brain Center Rudolf Magnus, University Medical Center Utrecht, Utrecht, the Netherlands; 2 Memory Aging & Cognition Centre, National University Health System, Singapore, Singapore; 3 Department of Pharmacology, National University of Singapore, Singapore, Singapore; 4 Image Sciences Institute, University Medical Center Utrecht, Utrecht, the Netherlands; 5 Academic Medicine Research Institute, Duke-NUS Graduate Medical School, National University of Singapore, Singapore, Singapore; 6 St. Luke’s Hospital, Singapore, Singapore; 7 Raffles Neuroscience Centre, Raffles Hospital, Singapore, Singapore; 8 Experimental Psychology, Helmholtz Institute, Utrecht University, Utrecht, the Netherlands; Nathan S Kline Institute, UNITED STATES

## Abstract

**Background and Purpose:**

Studies on the impact of small vessel disease (SVD) on cognition generally focus on white matter hyperintensity (WMH) volume. The extent to which WMH location relates to cognitive performance has received less attention, but is likely to be functionally important. We examined the relation between WMH location and cognition in a memory clinic cohort of patients with sporadic SVD.

**Methods:**

A total of 167 patients with SVD were recruited from memory clinics. Assumption-free region of interest-based analyses based on major white matter tracts and voxel-wise analyses were used to determine the association between WMH location and executive functioning, visuomotor speed and memory.

**Results:**

Region of interest-based analyses showed that WMHs located particularly within the anterior thalamic radiation and forceps minor were inversely associated with both executive functioning and visuomotor speed, independent of total WMH volume. Memory was significantly associated with WMH volume in the forceps minor, independent of total WMH volume. An independent assumption-free voxel-wise analysis identified strategic voxels in these same tracts. Region of interest-based analyses showed that WMH volume within the anterior thalamic radiation explained 6.8% of variance in executive functioning, compared to 3.9% for total WMH volume; WMH volume within the forceps minor explained 4.6% of variance in visuomotor speed and 4.2% of variance in memory, compared to 1.8% and 1.3% respectively for total WMH volume.

**Conclusions:**

Our findings identify the anterior thalamic radiation and forceps minor as strategic white matter tracts in which WMHs are most strongly associated with cognitive impairment in memory clinic patients with SVD. WMH volumes in individual tracts explained more variance in cognition than total WMH burden, emphasizing the importance of lesion location when addressing the functional consequences of WMHs.

## Introduction

Cerebral small vessel disease (SVD) is a major cause of cognitive impairment, either by itself or in combination with other pathologies in particular Alzheimer’s disease (AD) [1]. White matter hyperintensities (WMHs) are a major manifestation of SVD. Associations between WMH volume and cognitive functioning have been clearly established, but do show marked inter-individual variation [1,2]. Recent studies have highlighted the importance of WMH location in relation to cognitive functioning [3–8], which is consistent with the concept that disruption of strategic white matter tracts is a key mechanism through which subcortical vascular damage affects cognition [2]. Identifying strategic white matter tracts in which WMHs have most impact on cognition would improve our understanding of the functional impact of SVD.

Current image analysis techniques allow integration of three-dimensional information of lesion location in individual patients and anatomical knowledge of major white matter tracts in relation to cognitive performance. Recent studies have applied these techniques to identify strategic WMH locations in patients with CADASIL (cerebral autosomal dominant arteriopathy with subcortical infarcts and leukoencephalopathy), i.e. a rare form of hereditary SVD) [4,5], patients with manifest arterial disease [3], elderly stroke survivors (≥3 months post-stroke) [9], patients with AD [10] and community-based cohorts (i.e. patients without cognitive complaints) [6,7,11]. These studies suggest that WMHs located in frontal cortical-subcortical and frontal interhemispheric projections are associated with poor executive functioning and processing speed, while WMHs located in temporal and parieto-occipital white matter regions and the forceps major are associated with memory impairment. Furthermore, higher WMH volume within cholinergic projections has been associated with poor memory in AD patients [12]. However, tract-specific techniques have not yet been applied to memory clinic patient with sporadic SVD, a population in which an evaluation of functional impact of SVD is clearly clinically relevant, also in the context of other co-occurring etiologies, in particular AD. In the current study in memory clinic patients with sporadic SVD, we therefore aimed to (1) determine whether the impact of WMHs on cognition depends on location, and to (2) identify strategic white matter tracts in which WMHs have high impact on cognitive functioning.

## Materials and Methods

### Study subjects

Patients with SVD on MRI were selected from the National University Health System Memory Ageing and Cognition Centre cohort. Patients in this cohort were recruited from two memory clinics in Singapore (National University Hospital and St. Luke’s Hospital) between December 2010 and September 2013. Patients with the following five referral diagnoses were eligible for inclusion in the National University Health System Memory Ageing and Cognition Centre cohort: 1) Cognitive impairment not dementia: impairment in at least one cognitive domain, but without meeting the DSM-IV (Diagnostic and Statistical Manual of Mental Disorders–Fourth Edition) criteria for dementia. A domain was considered to be impaired if subjects failed at least half of the tests (i.e. a score more than 1.5 SDs below established normal means on individual tests, after adjustment for age and education) in that domain. 2) Alzheimer’s disease (AD): diagnosed using the NINCDS-ADRDA criteria for probable or possible AD [13]. 3) Vascular dementia (VaD): diagnosed using the NINDS-AIREN criteria [14]. 4) Mixed dementia: defined as AD with significant cerebrovascular disease using the McKhann criteria [13]. 5) No cognitive impairment: no objective cognitive impairment on formal neuropsychological tests, or functional loss. Patients with other diagnoses, significant neurological co-morbidities (e.g. Parkinson’s disease) or loss of functional independence (modified Rankin Scale >4) were not included in the cohort. All participants in the National University Health System Memory Ageing and Cognition Centre cohort underwent standardized clinical examination, neuropsychological testing (separate and independent from the primary testing at the memory clinics prior to study enrollment) and 3T MRI of the brain at the National University Hospital of Singapore.

**Fig 1:**
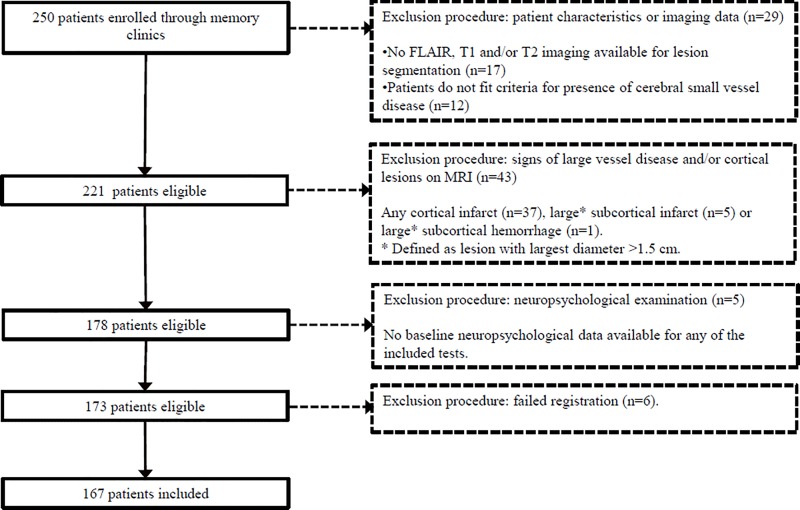
Flowchart of patient enrollment.

A flow chart of patient enrollment for the present study is provided in Fig 1. Of all 250 consecutive patients with the abovementioned referral diagnoses, only patients with complete baseline MRI sequences (T1, T2 and T2 FLAIR) were eligible for the present study (n = 233). Next, we selected all patients who met the following operational definition of presence of cerebral SVD: 1) MRI showing either moderate to severe WMHs (Fazekas ≥2), lacunes, infarcts, or intracerebral microbleeds (n = 205) [15]; or 2) MRI showing mild white matter lesions (Fazekas = 1) and at least two of the following vascular risk factors: hypertension, hypercholesterolemia, diabetes mellitus, obesity (BMI >30), past or current smoking and clinically manifest arterial disease (n = 16). Of these 221 patients, we excluded 43 patients with a cortical infarct or a large subcortical infarct (>15 mm) or hemorrhage (>15 mm), because such lesions can result in the complete obliteration of white matter tracts, thereby interfering with our analyses in which WMH volume within specific tracts is related to cognition at a group level (see analysis section). Five additional patients were excluded due to missing neuropsychological data for one or more of the tests for executive functioning, visuomotor speed or memory. Finally, 6 patients were excluded due to failed lesion registration. This resulted in a final study sample of 167 patients.

### Standard protocol approvals and patient consents

Ethical approval for this study was obtained from the National Healthcare Group Domain-Specific Review Board (DSRB). The study was conducted in accordance with the Declaration of Helsinki. Written informed consent was obtained by bilingual study coordinators in the preferred language of the patients. Informed consent was obtained face to face with the participant and an authorized family member present. No separate consent forms were used for cognitively impaired person; in such cases, demented participants indicated their willingness to join verbally. Legally acceptable representatives were approached to give consent on behalf of the individuals judged incapable of providing consent (e.g. severely demented patients). All queries were answered and clarified prior to the signing of the document to ensure that participants and family members had their concerns satisfactorily addressed.

### MRI protocol

MR investigations were performed on a 3 tesla Siemens Magnetom Trio Tim system, with a 32-channel head-coil, at the Clinical Imaging Research Centre of the National University of Singapore. The standardized protocol consisted of a 3D T1-weighted (1.0x1.0x1.0 mm^3^ voxels; TR 2300 ms; TE 1.9 ms; TI 900 ms; flip angle 9°; matrix 256x256), a 2D multislice T2-weighted (1.0x1.0x3.0 mm^3^ voxels; TR 3000 ms; TE 10.1 ms; matrix 247x256), a 2D multislice fluid-attenuated inversion recovery (FLAIR) (1.0x1.0x3.0 mm^3^; TR 9000 ms; TE 82 ms; TI 2500 ms; matrix 232x256), and a 2D multislice T2*-weighted image (1.0x1.0x1.5 mm^3^ voxels; TR 27 ms; TE 20 ms; flip angle 15°; matrix 192x256).

### Lesion segmentation

WMHs were manually segmented on T2 FLAIR sequences by a trained researcher (NAW) and reviewed by an experienced rater (JMB), who were blinded to the clinical data. The presence of infarcts and lacunes was initially scored independently by two raters (NAW and SH) and subsequently reviewed by another rater (JMB), followed by a consensus rating with another experienced rater (GJB) if there was uncertainty regarding lesion classification (which was the case in 23 patients (14%)). T1 and T2-weighted image sequences were used as reference to support proper lesion classification. Microbleeds were assessed by one rater (SH) on T2*-weighted images using the Brain Observer Micro-Bleeds Scale criteria [16]. The interrater and intrarater reliability as expressed by k-statistic ranged from 0.59 to 0.80 for lacunes, and 0.75 to 0.89 for microbleeds. All raters were blinded to the clinical data during the segmentation and rating process. All lesions were defined in accordance with the internationally established STRIVE criteria, which include guidelines on how to differentiate lacunes from perivascular spaces [17]. WMH segmentation was performed with in-house developed software based on MeVisLab (MeVis Medical Solutions AG, Bremen, Germany) [18,19]. Total brain volume and intracranial volume were quantified using the Statistical Parametric Mapping 12b unified segmentation approach [20] and used to compute the brain parenchymal fraction (BPF; (brain volume/intracranial volume)) as a measure of brain atrophy.

### Neuropsychological assessment

Our study focused on executive functioning and visuomotor speed, because these cognitive domains are commonly affected in patients with SVD [21], and on memory. The neuropsychological examination protocol and formation of compound scores for cognitive domains were hypothesis-driven and has been previously described [22]. Neuropsychological tests were administered in the patient’s habitual language to avoid variance due to insufficient language skills. Executive functioning was assessed with the Colour Trails Test 1&2 and verbal category fluency tests for animals and food [23,24]. Visuomotor speed was assessed with the Digit Cancellation Task, Symbol Digit Modalities Task and Maze Test [25–27]. Memory was assessed with verbal word list [28] and visual picture [29] recall and recognition tests. Z-scores were calculated for each subtest based on the mean and SD values of the included patients; results for the Maze Test and Colour Trails Test 1&2 were multiplied by -1, so that lower scores indicated poorer performance. Compound z-scores for executive functioning, visuomotor speed and memory were calculated by taking the average of the z-scores of the subtests for each cognitive domain.

### Generation of lesion maps

WMH segmentations were transformed to the T1 1-mm MNI-152 template using the following procedure [30]. All registrations were performed with the elastix toolbox for registration [31]. The results of the brain segmentation were used to create a brain tissue mask [20]. The T2 FLAIR scans were transformed to the corresponding T1 scans using a linear registration. The T1 scans were transformed to the Alzheimer’s disease (AD) T1 brain template [32], with a linear registration followed by a non-linear registration. For the linear registration, the brain tissue masks of both the patients’ T1 scan and the AD template were aligned to create a rough initial estimate and the original scans were used in the subsequent non-linear registration. The AD template was transformed to the MNI-152 template with a linear and a non-linear registration. The intermediate registration step using the AD template proved more successful in transforming scans of patients with severe atrophy, because of a better match between patient and template. Finally, all computed transformations were composed into a single transformation step that was used to co-register the WMH maps directly to the MNI-152 template. Composing transformations avoids any intermediate interpolations and results in a more accurate registration. Visual checks of the results of this procedure were performed for all patients. Six patients (4%) were excluded because the registration was unsuccessful.

### Statistical analyses

We performed our analyses in two subsequent steps. In the first step, we aimed to determine whether the impact of WMHs on cognitive functioning depends on location. In order to identify strategic white matter tracts in which WMHs are associated with cognitive functioning, independent of global WMH volume, we performed the following assumption-free (as opposed to hypothesis-driven) analyses: A) Region of interest (ROI)-based analyses based on the association between WMH volumes within specific white matter tracts and cognitive functioning, and B) voxel-based lesion-symptom mapping (VLSM) based on the association between the presence of WMHs and cognitive functioning for each affected voxel across the brain [33,34]. ROI-based analyses have the advantage of taking into account cumulative lesion burden within a white matter tract, whereas VLSM offers much higher spatial resolution (at the cost of lower statistical power due to more rigorous correction for multiple testing) without the need for predefining regions of interest. These two methods therefore have complementary strengths. For the ROI-based analyses, 11 regions of interest were created using a probabilistic white matter tract atlas with a probability threshold of 10% [35]. Bilateral tracts were merged into a single region of interest (for example, the anterior thalamic radiation was formed by merging the left and right anterior thalamic radiation into a single region of interest). The regional WMH volumes within these 11 white matter tracts were entered as independent variables in a linear regression model, with the z-scores of cognitive performance as outcome, and age, sex, level of education, total normalized WMH volume (i.e. WMH volume after registration to the MNI-152 template) and the presence of lacunes as covariates. These correlations were adjusted for total WMH volume, because strategic white matter tracts were defined as tracts in which WMH volume is correlated with cognitive functioning independent of global lesion volume. Next, VLSM analyses were performed using the compound z-scores for executive functioning and visuomotor speed, after individualized correction for age, sex and level of education using linear regression. To determine if the observed voxel-wise associations were independent of total lesion burden, the analyses were repeated after additional correction for total normalized WMH volume (i.e. WMH volume after normalization to the MNI-152 template) and the presence of lacunes. VLSM was performed using Non-Parametric Mapping (settings: univariate analysis, Brunner-Munzel test) [35]. Voxels that were damaged in less than 10 individuals were excluded from the analysis. Correction for multiple testing was done by applying a false discovery rate (FDR) with q<0.05. VLSM could not be performed for lacunes or microbleeds due to relatively low prevalence and small lesion size.

In the second step, we aimed to quantify and compare the impact of WMH lesion load in the strategic tracts as identified in the first step and other markers for SVD and brain injury, including total WMH burden on cognitive functioning and compare this to. For this purpose, ROI-based linear regression analyses were performed with cognitive functioning as the dependent variable, and the following measures separately entered as independent variables: global WMH volume, WMH volume within strategic white matter tracts (as identified in the first step), the presence of lacunes and microbleeds, and BPF. Finally, we performed a sensitivity analysis in which the ROI-based analyses were repeated after the exclusion of patients with AD.

## Results

Demographic and clinical characteristics of the study cohort are shown in Table 1. Forty-eight (28.7%) of the patients had one or more lacunes. WMHs were present in all patients. The cognitive profile of the study cohort is shown in Table 2.

**Table 1:** Characteristics of the study cohort.

Demographic characteristics	Study cohort (n = 167)
Age, mean (SD)	72.8 (9.1)
Male, n (%)	77 (46.1)
Education (years), mean (SD)	6.1 (5.0)
**Vascular risk factors, n (%)**	
Hypertension	128 (77)
Hyperlipidemia	119 (71)
Current smoker	16 (10)
Diabetes mellitus	73 (44)
History of stroke	27 (16)
**Imaging characteristics**	
Patients with lacunes, n (%)	48 (28.7)
Median WMH volume, ml (range) ^a^	14.7 (0.52–139.06)
Patients with microbleeds, n (%) ^b^	95 (56.9)
Brain Parenchymal Fraction, mean (SD)	0.64 (0.06)
**Referral diagnosis, n (%)**	
Alzheimer’s disease	42 (25.1)
No cognitive impairment	24 (14.4)
Cognitive impairment not dementia	62 (37.2)
Vascular dementia	7 (4.2)
Mixed dementia	32 (19.2)

^a^ Volumes after registration to MNI space (normalized volumes).

^b^ Data missing in four patients.

**Table 2:** Cognitive profile of the study cohort.

Neuropsychological test	Mean (SD)	Range
Mini-Mental State Examination (MMSE)	20.6 (6.2)	3–30
Montreal Cognitive Assessment (MoCA)	16.3 (7.2)	1–29
**Executive functioning**		
Verbal fluency animals (no. of words in 60 seconds)	10.0 (5.3)	0–23
Verbal fluency food (no. of words in 60 seconds)	10.8 (5.6)	0–32
Color trails test 1 (time in seconds, max 240)	148.3 (71.3)	32–240
Color trails test 2 (time in seconds, max 240)	198.3 (52.4)	66–240
**Visuomotor speed**		
Digit cancellation task (no. correct minus no. incorrect)	14 (9)	0–40
Symbol digit modalities task (no. correct in 90 seconds, max 110)	17 (15)	0–59
Maze task (time in seconds, max 240)	115 (93)	12–240
**Memory**		
Wordlist, immediate recall (no. correct, max 30) ^a^	11.4 (5.8)	0–23
Wordlist, delayed recall (no. correct, max 10) ^a^	1.4 (2.2)	0–8
Wordlist, recognition (true positive minus false positive)	4.7 (3.1)	0–10
Picture, immediate recall (no. correct, max 30) ^a^	3.6 (2.2)	0–9
Picture, delayed recall (no. correct, max 10) ^a^	2.3 (2.3)	0–8
Picture, recognition (true positive minus false positive)	6.9 (3.4)	0–10

^a^ Time interval between immediate and delayed recall is 15 minutes.

### Step 1A: region of interest-based analyses

The aim of these assumption-free ROI-based analyses was to identify strategic white matter tracts in which WMHs are associated with cognitive functioning, independent of global lesion burden. The results are shown in Table 3. First, age, sex and level of education were entered as variables in the linear regression model. Model 1 shows that these variables—as expected—explained a large proportion of the variation for executive functioning (R^2^ = 37.0%, p<0.001), visuomotor speed (R^2^ = 49.8%, p<0.001) and memory (R^2^ = 31.2%, p<0.001). Second, presence of lacunes and total WMH volume were added to model 1 (model 2). Next, WMH volumes within the 11 white matter tracts were each separately added to model 2 (models 3a-k). WMH volume in the anterior thalamic radiation and forceps minor was significantly associated with both executive functioning and visuomotor speed, independent of total WMH volume and presence of lacunes. WMH volume within the forceps minor was significantly associated with memory, independent of total WMH volume and lacunes, but WMH volume within the anterior thalamic radiation was not. Correlations between WMH volume within the remaining nine tracts and cognitive functioning were not statistically significant.

**Table 3:** Relation between WMH volume within 11 white matter tracts and executive functioning, visuomotor speed and memory.

		Executive		Speed		Memory	
Model	Independent variables	R^2^	p-value R^2^	R^2^	p-value R^2^	R^2^	p-value R^2^
1	Age, sex and years of education	0.370	<0.001*	0.498	<0.001*	0.312	<0.001*
2	Model 1 + presence of lacunes and total WMH volume	0.417	0.002*	0.517	0.046	0.341	0.030
3a	Model 2 + WMH volume Forceps minor	0.450	0.002*	0.547	0.001*	0.385	0.001*
3b	Model 2 + WMH volume Anterior thalamic radiation	0.455	0.001*	0.545	0.002*	0.368	0.010
3c	Model 2 + WMH volume Uncinate fasciculus	0.444	0.006	0.539	0.006	0.363	0.020
3d	Model 2 + WMH volume Inferior fronto-occipital fasciculus	0.436	0.020	0.527	0.061	0.354	0.072
3e	Model 2 + WMH volume Cingulum of cingulate gyrus	0.430	0.052	0.522	0.175	0.362	0.023
3f	Model 2 + WMH volume Inferior longitudinal fasciculus	0.420	0.371	0.517	0.654	0.342	0.565
3g	Model 2 + WMH volume Corticospinal tract	0.419	0.473	0.517	0.980	0.357	0.044
3h	Model 2 + WMH volume Forceps major	0.418	0.619	0.517	0.934	0.341	0.964
3i	Model 2 + WMH volume Superior longitudinal fasciculus	0.446	0.004	0.536	0.011	0.372	0.005
3j	Model 2 + WMH volume Temporal part of SLF	0.435	0.022	0.533	0.018	0.365	0.015
3k	Model 2 + WMH volume Cingulum of hippocampus	0.418	0.487	0.518	0.495	0.342	0.537

This assumption-free ROI-based analysis served to identify strategic white matter tracts in which WMH volume is correlated with executive functioning, visuomotor speed or memory, independent of total lesion burden. For this purpose, we first entered age, sex and education (model 1), and then total WMH volume and lacunes (model 2). Subsequently, all regional WMH volumes were entered separately (models 3a-k) to identify strategic white matter tracts in which regional WMH volume explained additional variance in cognitive functioning on top of total lesion burden. The p-value applies to the difference in explained variance (Δ R^2^) between each model and the previous model. SLF: Superior longitudinal fasciculus.

* = Statistically significant after Bonferroni correction for 13 comparisons (corresponding with p-value < 0.0038).

### Step 1B: voxel-based lesion-symptom mapping

**Fig 2:**
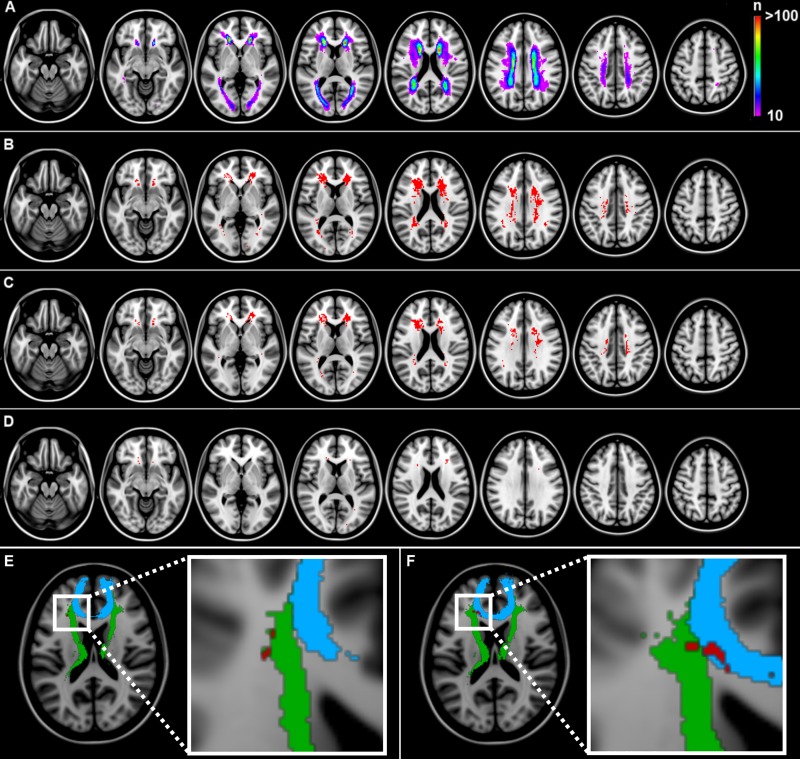
Lesion prevalence map and voxel-based lesion-symptom mapping results. (A) Lesion prevalence map. Voxels with white matter lesions in at least 10 patients are projected onto the MNI-152 template. (B-C): VLSM results for executive functioning (panel B), visuomotor speed (panel C) and memory (panel D) after correction for age, sex, level of education and multiple testing (settings: BM-test; FDR q<0.05). Significant voxels are shown in red. Z-coordinates: -20, -10, 0, 10, 20, 30, 40, 50. (E-F) VLSM results for executive functioning (panel E; coordinate Z-17) and visuomotor speed (panel F; coordinate Z-16) after additional correction for total WMH volume and presence of lacunes. One representative slice is shown per cognitive domain that depicts the location of several significant voxels (in red) in relation to the forceps minor (blue) and anterior thalamic radiation (green). The location of all significant voxels are provided in Table 4.

The aim of this assumption-free voxel-wise analysis was to identify strategic voxels (as opposed to white matter tracts in step 1A) in which WMHs are associated with cognitive functioning, independent of global lesion burden. The spatial distribution of WMHs is displayed in the lesion prevalence map in Fig 2 (panel A). As expected, WMHs were most prevalent in periventricular and frontal white matter regions. The results of the VLSM analyses on executive functioning, visuomotor speed and memory are shown in Fig 2 (panels B-C). VLSM identified a large number of voxels with a significant association between the presence of WMHs and poor executive functioning, visuomotor speed and memory after correction for age, sex, level of education and multiple testing. Several of these voxels remained significant after additional correction for total WMH volume and the presence of lacunes (panels D-E of Fig 2). These voxels were almost exclusively located within the frontal subcortical white matter, mostly within the anterior thalamic radiation and forceps minor (Table 4).

**Table 4:** Voxel-based lesion-symptom mapping results: tested and significant voxels for each anatomical region, after additional correction for total WMH volume and the presence of lacunes.

Anatomical regions (JHU atlas)	Region size in voxels (n)	Tested voxels (n)	Significant voxels (n)		
			executive	speed	memory
Forceps minor	34672	4125	6	27	2
Anterior thalamic radiation	42615	10291	8	48	2
Uncinate fasciculus	14203	3029	6	25	4
Inferior fronto-occipital fasciculus	48651	15183	6	40	1
Cingulum of the cingulate cortex	12870	533	0	0	0
Inferior longitudinal fasciculus	37133	5423	0	0	0
Corticospinal tract	27593	5136	0	0	0
Forceps major	22076	9805	2	0	0
Superior longitudinal fasciculus	59437	10301	0	0	0
Superior longitudinal fasciculus (temporal part)	24447	4470	0	0	0
Cingulum of the hippocampus	4632	1	0	0	0

### Step 2: quantification of the impact of WMHs in strategic tracts and other markers for SVD on executive functioning, visuomotor speed and memory

The explained variance in executive functioning, visuomotor speed and memory for each of the different imaging markers is provided in Table 5. WMH volume within the anterior thalamic radiation explained 6.8% of variance in executive functioning on top of age, sex and level of education (p<0.001), compared to 3.9% for total WMH volume (p = 0.001; on average only 11% of total WMH volume was contained in the anterior thalamic radiation). WMH volume within the forceps minor explained 4.6% of variance in visuomotor speed on top of age, sex and level of education (p<0.001), compared to 1.8% for total WMH volume (p = 0.013; on average only 4% of total WMHs were located within the forceps minor). WMH volume within the forceps minor explained 4.2% (p = 0.001) of variance in memory on top of age, sex and level of education (p<0.001), compared to 1.3% (p = 0.072) for total WMH volume. BPF explained 18.4% of variance in executive functioning (p<0.001), 11.7% of variance in visuomotor speed (p<0.001), and 15.6% of variance in memory (p<0.001) on top of age, sex and education. In a multivariable linear regression analysis, BPF was strongly correlated with WMH volume within the anterior thalamic radiation (ß -0.21, 95%CI -0.34 to -0.08; p = 0.002) and forceps minor (ß -0.23, 95%CI -0.36 to -0.11; p<0,001), independent of age, sex, education, lacunes and microbleeds, whereas total WMH volume was not (ß -0.04, 95%CI -0.17 to 0.09; p = 0.574). After adjustment for BPF, the correlations between total WMH volume, WMH volume within the anterior thalamic radiation and forceps minor, and executive functioning and visuomotor speed were attenuated, but remained statistically significant (Table 5), whereas the correlations between total WMH volume and WMH volume within the forceps minor and memory were no longer statistically significant. Lacunes (ß 0.03, 95%CI -0.10 to 0.16 for executive functioning; ß -0.02, 95%CI -0.14 to 0.09 for visuomotor speed; ß 0.10, 95%CI -0.04 to 0.23 for memory) and microbleeds (ß -0.04, 95%CI -0.16 to 0.09 for executive functioning; ß 0.00, 95%CI -0.11 to 0.12 for visuomotor speed; ß 0.00, 95%CI -0.13 to 0.13 for memory) were not significantly associated with cognitive functioning. In the sensitivity analysis, in which the ROI-based analyses were restricted to 125 patients without a diagnosis of AD, the main results were essentially the same (Table 6).

**Table 5:** Comparison of the impact of total WMH volume, regional WMH volume and brain atrophy on executive functioning, visuomotor speed and memory.

		Executive functioning			Visuomotor speed			Memory		
Model	Independent variables	R^2^	p-value R^2^	Beta (95% CI)	R^2^	p-value R^2^	Beta (95% CI)	R^2^	p-value R^2^	Beta (95% CI)
1	Age, sex and years of education	0.370	<0.001*	-	0.498	<0.001*	-	0.312	<0.001*	-
2a	Model 1 + total WMH volume	0.409	0.001*	-0.20 (-0.33 to -0.08)	0.516	0.013*	-0.14 (-0.25 to -0.03)	0.325	0.072	-0.12 (-0.25 to 0.01)
2b	Model 1 + WMH volume Fmin	0.435	<0.001*	-0.26 (-0.38 to -0.14)	0.544	<0.001*	-0.22 (-0.32 to -0.1)	0.354	0.001*	-0.21 (-0.34 to -0.08)
2c	Model 1 + WMH volume ATR	0.438	<0.001*	-0.27 (-0.39 to -0.15)	0.538	<0.001*	-0.21 (-0.32 to -0.10)	0.340	0.009*	-0.18 (-0.31 to -0.05)
2d	Model 1 + brain atrophy (BPF)	0.554	<0.001*	0.55 (0.42 to 0.68)	0.615	<0.001*	0.44 (0.31 to 0.56)	0.468	<0.001*	0.50 (0.36 to 0.65)
3a	Model 2d + total WMH volume	0.580	0.002*	-0.17 (-0.27 to -0.06)	0.627	0.029*	-0.11 (-21 to -0.01)	0.475	0.156	-0.08 (-0.20 to 0.03)
3b	Model 2d + WMH volume Fmin	0.571	0.015*	-0.11 (-0.19 to -0.02)	0.628	0.021*	-0.09 (-0.17 to -0.01)	0.476	0.129	-0.09 (-0.21 to 0.03)
3c	Model 2d + WMH volume ATR	0.576	0.004*	-0.07 (-0.11 to -0.02)	0.628	0.022*	-0.05 (-0.09 to -0.01)	0.472	0.271	-0.07 (-0.19 to 0.05)

The explained variance (R^2^) in executive functioning, visuomotor speed and memory is given for total WMH volume and WMH volume within strategic white matter tracts (the anterior thalamic radiation and forceps minor; model 2a-c). As a frame of reference, the explained variance for brain atrophy is also provided (models 2d and 3a-c). The p-value applies to the difference in explained variance (Δ R^2^) between the model and the previous model. Standardized coefficients (Beta) with corresponding 95% CIs are provided. BPF: brain parenchymal fraction. WMH: white matter hyperintensity. Fmin: Forceps minor. ATR: Anterior thalamic radiation.

* Statistically significant.

**Table 6:** Sensitivity analysis, restricted to patients without a diagnosis of AD (n = 125).

		Executive functioning			Visuomotor speed			Memory		
Model	Independent variables	R^2^	p-value R^2^	Beta (95% CI)	R^2^	p-value R^2^	Beta (95% CI)	R^2^	p-value R^2^	Beta (95% CI)
1	Age, sex and years of education	0.408	<0.001*	-	0.507	<0.001*	-	0.353	<0.001*	-
2a	Model 1 + total WMH volume	0.502	<0.001*	0.32 (-0.45 to -0.19)	0.552	<0.001*	-0.22 (-0.34 to -0.09)	0.407	0.001*	-0.24 (-0.38 to -0.10
2b	Model 1 + WMH volume Fmin	0.523	<0.001*^a^	-0.35 (-0.48 to -0.22)	0.582	<0.001*^a^	-0.28 (-0.40 to -0.16)	0.449	<0.001*^a^	-0.32 (-0.46 to -0.18)
2c	Model 1 + WMH volume ATR	0.525	<0.001*^a^	-0.36 (-0.50 to -0.23)	0.569	<0.001*^a^	-0.26 (-0.39 to -0.14)	0.422	<0.001*^a^	-0.28 (-0.42 to -0.13)

Sensitivity analysis of patients without a diagnosis of AD. The explained variance (R^2^) in executive functioning, visuomotor speed and memory is given for each linear regression model with the corresponding p-value for the difference in explained variance (Δ R^2^) between the model and the previous model. Standardized coefficients (Beta) with corresponding 95% CIs are provided. BPF: brain parenchymal fraction. WMH: white matter hyperintensity.

^a^ The correlations between WMH volumes in this tract and cognitive functioning remained significant after additional adjustment for total WMH volume (data not shown). Fmin: Forceps minor. ATR: Anterior thalamic radiation.

* Statistically significant.

## Discussion

Our findings identify the anterior thalamic radiation and forceps minor as strategic white matter tracts in which WMHs are most strongly associated with cognitive functioning in memory clinic patients with SVD. Importantly, these tracts were identified through corroborative evidence from two statistically independent analysis methods: ROI-based regression analyses and non-parametric voxel-wise analyses. The relevance of WMH location is further emphasized by the results of the ROI-based regression analyses (Table 5), which demonstrate that WMH volume within these white matter tracts explains two to three times more variance in cognition than total WMH volume. This confirms that the impact of WMHs on cognitive functioning depends on location, and indicates that strategically located WMHs have far greater impact on executive functioning, visuomotor speed and memory than global WMH volume in memory clinic patients with sporadic SVD.

An inverse association between total WMH volume and cognitive performance has previously been clearly established at a group level [1,2,36]. However, it remains difficult to determine to what extent WMHs are the cause of cognitive impairment for an individual patient, because WMH burden is clearly not linearly linked to cognition at an individual level. Furthermore, the amount of variance in cognition in memory clinic patients and non-demented older individuals with SVD that is explained by total WMH volume is low, generally in the order of a few percent [3,37,38]. Therefore, the assessment of whether and to what extent clinical symptoms are caused by WMHs is often based on the clinician’s expert judgment [2]. As total WMH volume can only explain a limited proportion of the inter-individual variability in cognitive functioning, it has been suggested that the location of WMHs might be more relevant than total WMH volume. This concept has been supported by previous lesion-symptom mapping studies in patients with CADASIL [4,5], cognitively preserved patients with manifest arterial disease [3], elderly stroke survivors [9], patients with AD [10] and a community-based cohort [6,7]. However, these studies have focused on patients without frank cognitive impairment, patients with a rare genetic cause of SVD, elderly stroke survivors or patients with AD. The domain of older patients with cognitive complaints and sporadic SVD in the memory clinic has—to our knowledge—thus far never been studied. As such, our study is the first to demonstrate a crucial role of strategically located WMHs in this clinically highly relevant population.

It should be noted that we chose to address the spectrum of patients with SVD in a memory clinic setting, rather than limiting our study to patients with a specific clinical diagnosis such as vascular dementia or AD. The rationale behind this is that in a memory clinic setting, SVD most commonly co-occurs with other pathologies [1]. Hence, the impact of SVD on cognition in the setting of a memory clinic should, in our view, be addressed in the context of these other pathologies. We therefore included a representative cohort of patients with different levels of cognitive impairment with SVD in the context of different clinical diagnoses. This inclusion procedure served to ensure generalizability of our findings to patients who attend a memory clinic and have at least a minimal burden of SVD, regardless of the final diagnosis. Evidently, considering the impact of WMHs in the setting of other SVD and non-SVD etiologies required us to address other pathologies and imaging markers on cognitive performance. We performed a sensitivity analysis in which we excluded patients with AD. The results were essentially the same, although the impact of WMHs on cognition appeared to be even greater in this subset of patients (i.e. higher explained variance and standardized coefficients in the regression model), as might be expected when excluding patients who are diagnosed with dementia of non-vascular etiology. Brain atrophy, as measured by BPF, was the most relevant imaging marker in explaining variance in executive functioning (18.4%), visuomotor speed (11.7%) and memory (15.6%) independent of age, sex and education. This is not surprising considering that brain atrophy is a very robust marker for brain injury due to either SVD or Alzheimer pathology. The importance of brain atrophy as a marker for SVD is illustrated by a previous study in patients with CADASIL, a monogenic form of SVD, in which brain atrophy was more strongly associated with cognitive functioning than WMH and lacunar volume [4,39]. Of note, the mean age of these CADASIL patients was 48 years, suggesting that these patients had 'pure' SVD, without co-occurring AD [4]. Our results indicate that in memory clinic patients with sporadic SVD, WMH volume (both global and within the anterior thalamic radiation and forceps minor) is of additional value in explaining variance in executive functioning and visuomotor speed independent of brain atrophy, though this was not the case for memory (Table 5). Of note, we also observed an interplay between WMH location and brain atrophy: WMH volume in the anterior thalamic radiation and forceps minor was strongly correlated with brain atrophy, independent of age, sex, education, microbleeds and lacunes, whereas global WMH volume was not. This explains our observation that the increase in explained variance in cognitive functioning was similar for global and regional WMH volumes when BPF was included in the model (Table 5). This interplay suggests that atrophy and WMHs in specific locations may be linked causally, which is in agreement with previous studies in AD patients in which an association between brain atrophy and WMHs located in the periventricular white matter and cholinergic pathways was found [10,12]. Whether this involves shared causal factors for WMHs and atrophy (i.e. certain processes causing both atrophy and WMHs in particular locations) or neurodegeneration secondary to WMHs remains to be determined. Of note, previous studies have shown that damage to subcortical fiber tracts causes secondary cortical neurodegeneration and focal thinning of connected cortices [40]. In general, our findings underline the importance of taking lesion location into account when studying the role of SVD in cognitive decline and dementia, either alone or in combination with neurodegeneration. Further studies, particularly longitudinal, are needed to determine the relation between regional WMH volumes and other MRI markers for cognitive decline and dementia, their prognostic value, and whether regional WMH volumes have any value in predicting response to pharmacological treatment.

The present study has several strengths. First, we applied an extensive, standardized neuropsychological test battery and MRI scan protocol. Second, we included a substantial number of memory clinic patients with a high level of cognitive dysfunction and high WMH burden and, consequently, could include a large number of white matter tracts in our analyses. Finally, we have used two independent hypothesis-free methods (ROI-based linear regression models and non-parametric VLSM) that both identified the anterior thalamic radiation and forceps minor as strategic white matter tracts, thereby providing converging evidence. An additional benefit of combining these methods is that they have complementary strengths as VLSM provides very high spatial resolution (association per voxel), while tract-based analyses take into account the cumulative burden of lesions in a specific tract. There are also several potential limitations. First, we were unable to assess the potential influence of lacune and microbleed location on cognitive performance in this study. Despite the fact that these lesions were quite common, their small volume did not permit lesion-symptom mapping. Second, we have not analyzed potential lateralized functions of white matters tracts, since we merged bilateral tracts for the tract-based analyses. Previous studies, including some lesion-symptom mapping studies on the cognitive impact of vascular lesions, suggest that the left hemisphere plays a prominent role in processing speed tasks [5]. In our VLSM analyses (after correction for age, sex, education, lacunes and total WMH volume), we observed no clear lateralization of significant voxels for executive functioning, visuomotor speed or memory. Third, the scan of each individual had to be registered to the MNI-152 template in order to perform VLSM. This brain template is based on the brains of young healthy subjects. The degree of deformation of an individual’s brain depends on innate brain size and acquired atrophy. In subjects with an innate smaller brain or severe atrophy (in which case the white matter tracts will be more densely packed), the brain and WMHs are proportionally enlarged to match the template during registration. In order to optimize the quality of the registration procedure for patients with severe atrophy, we have used an Alzheimer atlas as intermediate step in the registration procedure, followed by registration to the MNI atlas. Fourth, WMH due to sporadic SVD only rarely affect the temporal regions: only four patients had WMH in the cingulum of the hippocampus, whereas all 167 patients had a certain amount of WMH in the forceps minor. This explains why no association was found between WMH and memory in the cingulum of the hippocampus even though this tract is known to be crucially involved in memory processes. Fifth, we did not distinguish between specific etiologies underlying the WMHs, such as cerebral amyloid angiopathy (CAA) or hypertensive vasculopathy [41]. Of note, the prevalence of microbleeds in our cohort is higher than generally reported in patients with AD and the general elderly population [42–44], but it should be noted that this is also due to the inclusion criteria for the current study, which focused on memory clinic patients manifesting small vessel disease. Finally, it would be interesting to apply a multi-level analysis in which markers of Alzheimer pathology and measures of microstructural integrity are integrated with data on the presence and location of vascular lesions. However, these data were not available for all patients and such an approach might require an even larger study sample.

## Conclusions

The current study identifies the anterior thalamic radiation and forceps minor as strategic white matter tracts in which WMHs are most strongly associated with executive functioning, visuomotor speed and memory performance in memory clinic patients with SVD. Our findings underline the concept that WMHs located in crucial white matter tracts most strongly affect cognition. Therefore, future studies on the pathogenesis of vascular cognitive impairment and the role of sporadic SVD in dementia should not only focus on total WMH volume, but should also take WMH location into account.

## Supporting Information

S1 File This file contains the data used in the linear regression analyses. (SAV)
